# Mechanisms of Gene Duplication and Translocation and Progress towards Understanding Their Relative Contributions to Animal Genome Evolution

**DOI:** 10.1155/2012/846421

**Published:** 2012-08-07

**Authors:** Olivia Mendivil Ramos, David E. K. Ferrier

**Affiliations:** The Scottish Oceans Institute, School of Biology, University of St Andrews, East Sands, Fife KY16 8LB, UK

## Abstract

Duplication of genetic material is clearly a major route to genetic change, with consequences for both evolution and disease. A variety of forms and mechanisms of duplication are recognised, operating across the scales of a few base pairs upto entire genomes. With the ever-increasing amounts of gene and genome sequence data that are becoming available, our understanding of the extent of duplication is greatly improving, both in terms of the scales of duplication events as well as their rates of occurrence. An accurate understanding of these processes is vital if we are to properly understand important events in evolution as well as mechanisms operating at the level of genome organisation. Here we will focus on duplication in animal genomes and how the duplicated sequences are distributed, with the aim of maintaining a focus on principles of evolution and organisation that are most directly applicable to the shaping of our own genome.

## 1. Introduction

**Figure 1:**
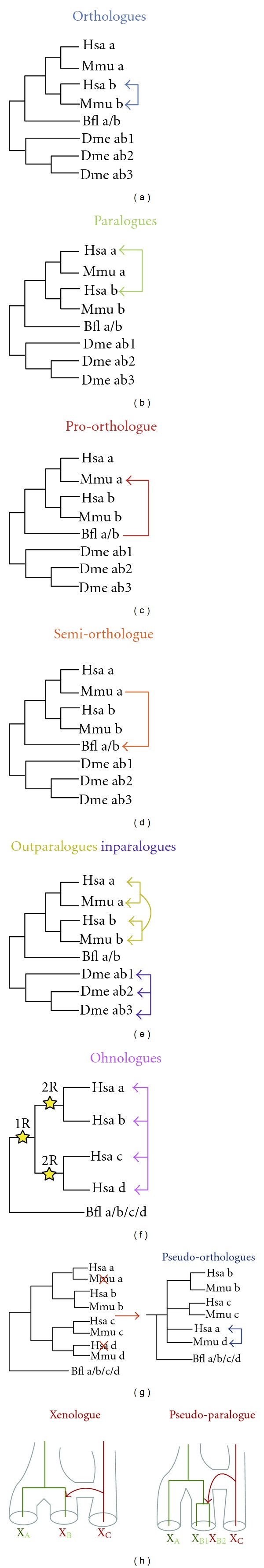
Overview of the current terminology. The different panels represent term(s) for duplicated genes. (a) Orthologues. The square blue arrows represent an orthologous relationship between the two genes. (b) Paralogues. The square green arrows represent paralogous relationships between the genes. (c) Proto-orthologue. The square red arrow represents the pro-orthologue relationship of gene a/b from *Branchiostoma floridae* to gene a from *Mus musculus*. (d) Semi-orthologue. The square orange arrow represents the semi-orthologous relationship of gene a of *Mus musculus* to gene a/b from *Branchiostoma floridae*. (e) Inparalogues and Outparalogues. The square yellow arrows represent the outparalogous relationship in which human and mouse a genes are outparalogous to human and mouse b genes. As a set, genes a and b from mouse and human represents coorthologues. The square purple arrows represent the inparalogous relationship between the genes which duplicated within this lineage. (f) Ohnologues. The square pink arrows delimit all the paralogues coming from WGD and the stars represent the duplication events. (g) Pseudo-orthologues. The square navy arrows represent the pseudo-orthologues. The red Xs represent lineage-specific gene losses. (h) Xenologues and Pseudo-paralogues. Species are represented by subindices A, B, and C, and the Xs represent the orthologous genes with their colouring designating the species of origin. All of the figures are adapted from [4–6]. Bfl: *Branchiostoma floridae*, Dme: *Drosophila melanogaster,* Hsa: *Homo sapiens*, and Mmu: *Mus musculus*.

New genes constitute some of the major raw material for the evolution of biodiversity. They do not arise out of thin air. Some instances of new gene evolution from previously non-coding sequence have now been discovered [1, 2]. Also, new genes can be formed by shuffling of pre-existing nucleotide sequences. The relatively recent discovery of large numbers of taxonomically restricted genes also demands a closer investigation of their mode(s) of origin [3]. Nevertheless, a major mechanism for the generation of new genes is via duplication. Such duplicates are called paralogues, to reflect their homologous relationship being due to a duplication event rather than a speciation event (see Figure 1).

 Since the first animal whole genome sequence of the nematode *Caenorhabditis elegans* [8], the number of animal whole genome sequences has been increasing at an impressive rate. It should, however, be kept in mind that there is a high level of variability in the “quality” of these genome sequences; “quality” here referring to the depth of sequence coverage of the genome, levels of effort to fill gaps in the sequence, and amount of independent mapping data to inform and confirm the assembly. As a result, many of the animal whole genome sequences that are available must be handled with caution when estimating the extent and nature of duplication events. Furthermore, most animal genome sequences can only be assembled to a subchromosomal scale, with genomic scaffolds covering only fragments of chromosomes. This becomes important when trying to assess duplication and translocation mechanisms and distinguishing intra- and interchromosomal events. Inevitably, the organisms with the largest research communities and the most intensively studied genomes tend to have the highest quality genome assemblies and annotations. Most studies of gene and genome duplications, and hypotheses about mechanisms, stem from analyses of such organisms as vertebrates (including humans, other mammals, and fish) and insect and nematode model systems, as will become clear below. 

Here we review the current terminology used for duplicated genes and then discuss the role of whole genome duplication, particularly within the context of vertebrate evolution, and review the current understanding of modes of subchromosomal duplications and recent data on mechanisms for distribution of these duplicated sequences around the genome.

## 2. Terminology: Beware Overlap, Synonyms, and Ambiguity (and Use with Care)

The terminology used to define the evolutionary relationships between duplicated genes has become increasingly detailed. The precise inference of the evolutionary relationships between duplicated genes is fundamental for most comparative genomic studies, but it can be complicated because duplication is often combined with speciation and subsequent gene loss [4].

The most widely used terms for describing evolutionary relationships between genes are homologous, orthologous, and paralogous. Fitch [9] defined homologous genes as those that share a common ancestor. A subset of homologous genes are orthologous, these being the genes separated only by speciation and not by a duplication event (Figure 1(a)). Another subset of homologous genes are paralogous, which are those resulting from a duplication event (Figure 1(b)). Sharman [4] defined additional terms to describe the relationships amongst paralogues. Pro-orthology denotes the relationship of a gene to one of the descendants of its orthologue after duplication of that orthologue (Figure 1(c)). Conversely, semi-orthology is the relationship of one of a set of duplicated genes to a gene that is orthologous to the ancestor of the whole set (Figure 1(d)). Sharman [4] also proposed the term trans-homology to describe members of the same gene family descendant from an ancestral gene via two independent gene duplication events. A further important term connected with paralogy is the one proposed by Wolfe [10], who coined the term ohnologue for those paralogues stemming from a whole genome duplication (Figure 1(f)). Two years later, Sonnhammer and Koonin [5] highlighted that the definition of a paralogous relationship can be related to a speciation event. Thus, they coined the terms inparalogues and outparalogues. Inparalogues are paralogues in a given lineage that all evolved by gene duplications that happened after a speciation event that separated the given lineage from the other lineage under consideration (Figure 1(e)). Outparalogues are paralogues in a given lineage that evolved by gene duplications that happened before a speciation event (Figure 1(e)). Careful consideration must be taken when using the terms such as inparalogues, outparalogues, and ohnologues. The specification of the relation of the duplication event to the speciation event must be included when these terms are used, otherwise evolutionary interpretations and use of terminology can easily be confused. Finally, a new umbrella term, duplogs [11], has been thrown into the duplication terminology pool to define intraspecies paralogues. This term amalgamates all the types of paralogues within a species, including inparalogues, outparalogues, and ohnologues. 

Sonnhammer and Koonin [5] also defined co-orthologues, which are synonymous with Sharman's [4] definition of trans-homologues, and are inparalogues of one lineage which are homologous to another set of inparalogues in a second lineage. Artifacts stemming from phylogenetic inference, such as lineage-specific gene loss, can mislead the deduction of the evolutionary relationship of genes. For this purpose, Koonin [6] devised the term pseudo-orthologue to accommodate those genes that are essentially paralogues but appear to be orthologues due to differential, lineage-specific gene loss (Figure 1(g)). Further useful terms are xenologue and pseudo-paralogue. Xenologues are homologues acquired through horizontal gene transfer by one or both species that are being compared, but appearing to be orthologues when pairwise comparison of the genomes is performed (Figure 1(h)) [6]. Pseudo-paralogues are homologues that through the analysis in a single genome are interpreted as paralogues; however, these homologues originated by a combination of vertical inheritance and horizontal gene transfer (Figure 1(h)) [6]. 

Recently a new term, toporthology, has been specified, which aims to include another aspect of the concept of orthology, that of positional orthology [12]. Toporthology describes the evolutionary relationship of orthologues that retain their ancestral genomic positions. In the context of gene duplications, a duplication event is said to be “symmetric” if deletion of either of the copies of the duplicated sequences would return the gene order to the original, ancestral state. Thus, tandem duplicates and whole-chromosome/genome duplication are symmetrical duplications. A duplication event is “asymmetric” if deleting only one of the copies could return the gene order to its original, ancestral state. Consequently, dispersed segmental duplications and retrotranspositions are asymmetrical duplications. From these definitions two genes are positionally homologous, topohomologous, if they are homologous and neither gene comes from an asymmetric duplication since the time of their common ancestor. The contrast to this case is atopohomologous. The topo- and atopo- prefixes can similarly be applied to orthologues and paralogues. 

The term toporthology and its associated derivations need to be used with extreme caution [12]. The value, and aim, of distinguishing toporthologues/topoparalogues is to distinguish those genes (which are not necessarily one-to-one orthologues) that are most comparable in terms of their evolutionary history. However, being able to distinguish toporthology obviously requires reliable, accurate genome assemblies and hinges on distinguishing parent/source locations from daughter/target locations of duplicated regions. Also, the distinction of toporthology can obviously be complicated by genomic rearrangements that occur after the duplication event and which can obscure whether a duplication was symmetric or asymmetric. Currently, the complications introduced by such postduplication genomic rearrangements lead to some counterintuitive uses of the terminology. One might assume that toporthology/topoparalogy simply refers to orthologues/paralogues that are both in the ancestral locations, and conversely that atoporthology/atopoparalogy simply describes the situation in which at least one of the genes is no longer in the ancestral location. The use of the terminology is not so straight-forward, however, as can be seen by a close inspection of Figure  2 in [12], in which YA1 and YA2 are topoparalogues rather than atopoparalogues despite YA2 no longer being in the ancestral location. The classification of YA1 and YA2 as topoparalogues arises because they were not produced by an asymmetric duplication, but then the subsequent change of position of YA2 has obscured this. Consequently the precision of the data (taxonomic sampling and quality of genome assembly) severely compromises the utility of this terminology. Despite the apparent use of the terms to reflect relationships relative to ancestral locations within the genome, in fact the movement of genes to new, nonancestral locations subsequent to the duplication event is not accommodated. Consequently toporthologues/topoparalogues are not necessarily both in the ancestral genomic position. This terminology thus risks being counterintuitive and confusing in its present form.

The above summary of duplicate terminology serves to illustrate two things. Firstly, there is the complexity of the evolutionary processes involved in production of duplicates and the care that must thus be exercised when comparing genes between species. Secondly, there is currently an over-abundance of terminology, some of which is redundant and some of which is counterintuitive. It is to be hoped that with time the terminology will settle on a consensus of selected terms and those that are impractical or potentially misleading will be abandoned. We now turn from the terminology of gene duplication to the biological processes and evolutionary events.

## 3. Whole Genome Duplications (WGDs): Origin of Vertebrates and 2R

One of the most striking features of the human genome, which is shared with the other members of our subphylum, the Vertebrata, is the extensive occurrence of paralogons: homologous regions of chromosomes that are related via duplication events rather than speciation events [13]. This observation is usually attributed to the occurrence of two rounds of whole genome duplication at the origin of the vertebrates (the so-called 2R hypothesis), because of the preponderance of four paralogons for each region of the human genome being considered. Thus, one copy of the diploid genome duplicated to give two copies, and this tetraploid state then duplicated a second time to effectively give an octoploid state [14], which with time has been “diploidized” again but with the remnants of the octoploid state being detectable from analyses of the paralogons. The 2R events were inevitably followed by extensive gene loss, as would be expected given the inevitable high levels of genetic redundancy that would ensue from such large-scale duplications, such that less than 30% of the 2R paralogous genes are estimated to remain [15]. This means that 2R paralogue families now consist of between two to four members [16], thus providing a significant pool of extra genes that have made a significant contribution to the evolution and diversification of the vertebrates.

This 2R hypothesis has its roots in the ideas of Susumu Ohno, and it then began to gain increasing support from molecular genetic work, principally from the invertebrate chordate amphioxus. For example, amphioxus has a single Hox gene cluster whilst humans have four [17, 18]. The 2R hypothesis was not universally accepted at first [19], largely on the grounds of differing interpretations of molecular phylogenetic trees and the assessment of branching topologies within different gene families and amongst paralogues. The topology argument that formed the basis for challenging the 2R hypothesis [19] requires the trees to be interpreted in a very restricted fashion, with the four paralogues adopting a symmetrical topology of ((A, B)(C, D)). This was supposed to represent the first WGD producing two paralogues, which were the precursors to AB and CD, followed by the second WGD producing the A and B as well as C and D genes. However, it is far from clear that duplicated genes always behave in the expected post-duplication way, with daughters evolving at equal rates post-duplication. In fact there is increasing evidence for asymmetric evolution of duplicated genes [20], often with disruptions to tree topology that tend to arise from Long Branch Attraction [21]. Also, as analyses progressed to genome-scale data the controversy has largely subsided with the ever-increasing evidence in favour of 2R. This is typified by the sequencing of the whole genome of the American amphioxus, *Branchiostoma floridae*, and analyses not just of paralogue phylogenies but also patterns of gene synteny across chordates. The trend for a single locus in amphioxus matching four loci in humans (and other vertebrates, with some notable exceptions mentioned below), which was originally developed from work on the Hox gene cluster(s) [22] was found to extend to large-scale, genome-wide Quadruple Conserved Synteny [23]. 

There have still been one or two dissenting voices, such as [24] arguing instead for segmental duplications occurring at different times rather than whole genome duplications (and hence simultaneous origins of paralogons). However, we note that the interpretation of the molecular phylogenies in [24] contains a number of errors, including deductions based on support values at inappropriate nodes as well as nodes that do not have significant support values. Questionable rooting strategies are employed in several of the trees in [24] and incomplete datasets are used for some genes, such as the Sp transcription factors [25]. The analyses of Abbasi [24] in fact do not challenge the 2R hypothesis, but in fact often support it as soon as one accepts that some gene loss occurred after 2R. That gene loss is a common phenomenon is now without doubt [15, 26–30]. Also, since both WGD events occurred close together in time, and via autotetraploidy in both cases, then it is to be expected that the phylogenies of the paralogues do not in fact adopt the ((A, B)(C, D)) topology, as explained by Furlong and Holland [14]. Tree topologies should thus not still be being used as a test of 2R with the view that divergence from the ((A, B)(C, D)) topology is in conflict with 2R. Furthermore, the 2R hypothesis no longer relies solely upon the topology of individual gene trees, but instead gains its most convincing support from conserved synteny arrangements that cover over 90% of the human genome and extends to the genomes of birds and fish (including chicken, stickleback, and puffer fish) [23]. Therefore, we hold the view that the 2R hypothesis (with subsequent gene loss) is definitely the most parsimonious explanation for the origin and evolution of vertebrate genomes.

The plausibility of the 2R hypothesis is further strengthened by the discoveries of whole genome duplications elsewhere in the animal kingdom, thus demonstrating that the process can certainly occur, and do so with reasonable frequency (see Table 1) [31, 32]. For example, the origin of the teleost fish coincides with another WGD, the 3R event. Again, this hypothesis is strongly supported by the patterns of synteny relative to other vertebrates and the existence of extensive paralogons matching the topology expected for a 3R event [33]. Whole genome duplications and polyploidization events are constantly coming to light within the animal kingdom, and are clearly a significant mode of duplication that has shaped animal evolution. Duplications also occur on a smaller scale, at the subchromosomal level.

## 4. Subchromosomal Duplications: Variable Sizes, Rates, and Mechanisms

Duplications that encompass sections of DNA smaller than whole chromosomes are given the generic name of segmental duplications (SDs). These can vary enormously in size, from a few base pairs up to many megabases, and may or may not contain intact, functional genes. They can also be found in several different arrangements, which are important for considerations as to how these SDs might form. SDs can be adjacent (tandem duplications), separated, or interspersed along a particular chromosome (intrachromosomal) or on distinct chromosomes (interchromosomal). The detection of SDs in these different categories obviously depends upon the quality of a genome sequence assembly, but the prevalence of SDs in the human genome, for example, tend to be estimated at about 5-6% (for SDs ≥1 kb, with ≥90% sequence identity, and filtered for transposable elements and other high-copy repeats) [62]. Estimates of SD prevalence in other mammals tends to produce slightly lower levels than in humans, although in the case of mouse that has recently been revised upwards to almost 5% and hence is now thought to be comparable to the levels in humans [62, 63]. A striking aspect of the comparisons between rates and distributions of SDs in various mammalian genome sequences is that tandem duplications are by far the most prevalent category of SD, comprising 75–90% of SDs in the cow for example [64]. This preponderance of tandem duplicates in mammals as diverse as cows, rodents, and dogs does not, however, reflect the situation in humans, in which SDs are much more frequently interspersed [64–67]. The interspersed distribution of human SDs is possibly the result of an expansion of Alu transposable elements within primates [62, 68]. Moving outside of the mammals, the fruit fly *Drosophila melanogaster* has the majority of its SDs in the intrachromosomal category (86%), and of these most are situated close together in the genome (50% and <14 kb apart) [69]. 

The different categories of SDs (tandem, interspersed intrachromosomal, and interchromosomal) may well reflect different mechanisms of DNA-based duplication. Non-homologous end-joining (NHEJ) is more likely to account for adjacent duplications [70–72] with the repair of DNA breaks being more likely to occur between ends in close proximity. The alternative of nonallelic homologous recombination (NAHR) is likely mediated via repetitive sequences dispersed around the genome and hence is a route to interspersed duplications. This process has been given the name duplication-dependent strand annealing (DDSA) by Fiston-Lavier et al. [69], who also noted that in *D. melanogaster* the mean size of intrachromosomal events is larger than the average size of interchromosomal events (3.1 kb versus 2.1 kb, respectively). This contrasts with the average size of SDs in humans being approximately 18.6 kb and 14.8 kb for the intrachromosomal and interchromosomal categories respectively [73].

In addition to this observation that intrachromosomal SDs tend to be longer than interchromosomal SDs possibly reflecting different mechanisms being the cause of their origin, it is striking that the size of SDs varies in different species. A further “data point” is provided by the nematode *Caenorhabditis elegans*, in which the average size of SDs is only 1.4 kb [74]. This implies that the size of duplication is not necessarily determined by physical properties of the DNA or possibly the duplication mechanism (unless mechanisms differ between the taxa thus far examined), but instead is likely to relate to the structure and organization of the genome. Density and distribution of repetitive sequences will be one factor, and these vary across different species. In addition, strong selective pressures are likely to come into operation when genes are duplicated within SDs, often disrupting genetic networks and pathways if a gene is duplicated and then expressed (e.g., via dosage imbalance [75]). Thus there will tend to be selective pressure against duplications that encompass genes (and their regulatory elements), thus reducing the average size of segmental duplicates in taxa with smaller, more compact genes.

Alongside consideration of the duplication mechanisms within the context of determining the organisation of duplicated genes, it follows that one must also consider processes by which segments of DNA or genes can be translocated around the genome. Although these mechanisms are not necessarily leading to generation of duplications (and in fact often are not) they are still crucial in understanding the subsequent distribution of genes, which in the present context happen to be duplicates. Retrotransposition is one of the duplication mechanisms that does not necessarily lead to generation of functional duplicated genes, but is crucial in distributing duplicated single genes, especially in an inter-chromosomal fashion [76–79]. Inversions are very common and help to scatter duplicated genes along a particular chromosome arm [71, 80]. Also large-scale events such as inversions between arms involving the centromere or chromosome fusions and fissions are also known to play a prominent role in karyotype evolution, and reciprocal translocations between chromosome arms are very common. Surprisingly high rates of reciprocal translocations occur in humans, with estimates of around one in 500 newborns carrying such large-scale rearrangements [81–84]. This is not necessarily unusual to humans, as cattle reciprocal translocations have been estimated to occur at a rate of 1.4 per 1000 animals [85]. These high rates of translocations are thought to be mediated via NAHR using duplicated or repetitive segments located in different chromosomes, that is interchromosomal low-copy repeats (LCRs) [86]. Ou et al. [86] characterized several hundred interchromosomal LCRs in the human genome, ranging in size from 5kb to over 50kb, all of which they suggest can act as the substrates for reciprocal translocations. In addition, Hermetz et al. [87] described a translocation occurring via homologous recombination between HERV elements on different chromosomes.

In combination all of these routes to rearrangement of genome organisation often make it difficult to accurately determine between likely mechanisms of duplicate origin. This is because it is difficult to determine whether the locations of any two duplicated sequences reflect their organisation at their point of origin, or instead is the end point of originating by a process such as tandem duplication and then subsequently being dispersed. Attempts to address this problem have involved estimating the age of duplicates by calculating the rates of synonymous substitutions (*K*_*s*_). This has led to observations that younger genes tend to be closer together in the genome, particularly being more highly represented in the intrachromosomal category of duplicates relative to the interchromosomal category [74, 88]. However, such estimates of gene age can be confounded by the process of gene conversion, which can homogenise gene sequence after the origin of the duplicates [89, 90]. Since gene conversion is more likely to occur between genes that are in close proximity then there will be a degree of misjudging the age of duplicates as inappropriately young, and this effect will be most pronounced in the categories of closely linked genes such as tandem duplicates. Furthermore, the positive correlation between age and dispersal in the genome has recently been questioned with the proposal of a process named drift duplication [11]. Ezawa and colleagues' [11] comparisons of duplicate age and genomic location in human, mouse, zebrafish, *C. elegans*, *D. melanogaster*, and *Drosophila pseudoobscura* suggest that interspersed intrachromosomal duplications can be generated at once, rather than originating as tandem duplicates which are subsequently relocated away from each other, and this can happen at comparable rates to tandem duplication [11].

**Figure 2:**
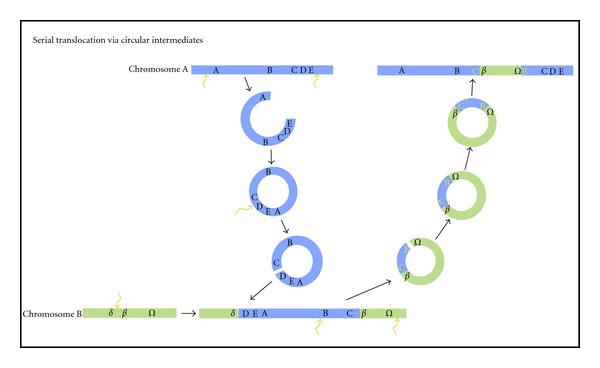
Scheme of a serial translocation via circular DNA intermediates. Two excisions create a fragment of chromosome A, delimited by genes A and E. This fragment circularizes. At reinsertion into a new genomic location, the circle is linearized by being opened between C and D and inserts between genes *∂* and *β* of chromosome B. The subsequent translocation involves an excision delimited by genes B and Ω. The fragment created circularizes and has sequence identity to the region on chromosome A between the C and B genes. This region of homology allows a repatriation of the segment of original genes from chromosome A, creating a duplication as well as translocating genes from chromosome B. Blue and green lines represent fragments of two different chromosomes. The capital and Greek letters represent genes within the chromosomes. The yellow capital letters denote the genes translocated from chromosome B (green line). The angled orange arrows represent excision points in the DNA. The orange cross represents a homologous recombination site. Adapted from [7].

The precise mechanism leading to drift duplication is not specified by Ezawa et al. [11], and is likely to involve a combination of processes. One of these could well be the recently discovered process of duplication via circular DNA-based translocation. Durkin et al. [7] recently found that in “lineback” or “witrik” cows a translocation of 492 kb occurred which was then followed by a repatriation of a 575 kb segment, including the KIT gene that is involved in the pigmentation patterning of the cows and their distinctive “lineback” phenotype. The intriguing aspect to these translocations is the order of sequences within the translocated segment, which is consistent with translocation via a circular DNA intermediate which is opened up for re-insertion at a different point in the circle from the boundaries of the original excision (Figure 2). Also, since the repatriated segment was larger than the originally translocated segment then some sequence duplication results (Figure 2). Further examples of duplications via circular DNA intermediates are being found, such as the vasa genes of *Tilapia* [91]. The difference between the cow and *Tilapia* examples however is that the cow circular DNA intermediate is repatriated into an ancestral locus, presumably due to homologous recombination, whereas the *Tilapia* vasa duplicates that arose via circular intermediates have gone to new locations. The *Tilapia* vasa example is thus more reminiscent of drift duplication, but it remains to be seen how prevalent such circular DNA translocation events are and how the reintegration sites are selected.

Given the range of genomic rearrangement mechanisms and their apparent frequencies, it is perhaps surprising that syntenic arrangements can be conserved for vast evolutionary timespans, for example, from humans to the origin of chordates [23] and beyond, to even some basal lineages of animals such as the cnidarian *Nematostella vectensis* and the placozoan *Trichoplax adhaerens* [92, 93]. What is also striking is that this phenomenon of long-term general synteny conservation is not detected uniformly across the animal kingdom. Some lineages and groups of animals seem to have particularly derived genome organisations relative to other animals (e.g., *Oikopleura* and urochordates in general; *Drosophila* and other Diptera; nematodes like *C.elegans* [8, 94, 95]). One could speculate that this might reflect different abundances of repetitive elements, for example, which can have a role in facilitating genomic rearrangements. Another possibility is that gene sizes, and perhaps more importantly gene densities within the chromosomes, vary significantly across the animal kingdom. This variation might not just be the number of nucleotides spanned by the coding sequence, but also by the regulatory elements, which will influence how frequently rearrangement mutations can occur that are still compatible with organismal viability. Regardless of this, some animal genomes seem to be more tolerant of, or prone to, rearrangements than others. With the burgeoning amounts of human genome sequence data, particularly in relation to disease and cancer genomics, a new phenomenon involving a catastrophic rearrangement of the genome has recently been described: chromothripsis [96, 97]. Perhaps the process of chromothripsis has a relevance beyond the realms of cancer and disease biology and may be comparable to processes whereby some animal genomes become extensively rearranged relative to other lineages.

## 5. Conclusion

Gene and genome duplication constitute major forces in evolutionary innovation. The variety of mechanisms by which such duplications occur, as well as the various means by which the duplicated segments are subsequently rearranged (and sometimes partially lost), requires careful analysis and consistent use of biologically informed terminology. Obviously a major goal for the future will be to expand the taxonomic coverage of high-quality genome assemblies to enable the deduction of more accurate and more widely applicable, general conclusions about such phenomena as gene and genome duplications. This should be complemented by the continued development of in silico tools and models to estimate duplication and rearrangement rates. Such tools then need to be applied across an increased range of genomes in order to distinguish general mechanisms and principles from lineage-specific oddities, such as lack of synteny between urochordates and vertebrates or the paucity of tandem duplications in humans relative to other mammals. 

## Figures and Tables

**Table 1:** Examples of species undergoing whole genome duplication or polyploidisation events. Adapted from [31, 32].

Species/Taxon (Common name)	References
*Xenopus laevis* (African clawed frog)	Morin et al. [34]
*Tympanoctomys barrerae* (red viscacha rat)	Gallardo et al. [35]
*Daphnia pulex* (water flea)	Vergilino et al. [36]
*Schmidtea polychroa* (planarian flatworm)	D'Souza et al. [37]
*Acipenser brevirostrum* (shortnose sturgeon)	Fontana et al. [38]
*Scaphirhynchus platorynchus* (shovelnose sturgeon)	Schultz [39]
*Polyodon spathula* (american paddlefish)	Schultz [39]
*Menidia* sp. (atlantic silverside)	Echelle and Mosier [40]
*Barbatula barbatula* (stone loach)	Collares-Pereira et al. [41]
*Catostomidae* (suckers)	Schultz [39]
*Botia* spp. (pakistani loach)	Yu et al. [42], Rishi and Shashikala Rishi [43]
*Cobitis* spp. (loach)	Schultz [39], Vrijenhoek et al. [44], Janko et al. [45]
*Misgurnus anguillicaudatus* (dojo loach)	Arai et al. [46]
*Misgurnus fossilis* (european weather loach)	Raicu and Taisescu [47]
*Barbodes* spp. (tinfoil)	Chenuil et al. [48]
*Barbus* spp. (barb)	Suzuki and Taki [49]
*Acrossocheilus sumatranus* (large-scale barb)	Suzuki and Taki [49]
*Aulopyge hugelii* (dalmatian barbelgudgeon)	Mazik et al. [50]
*Cyprinus carpio* (carp)	Wang et al. [51]
*Carassius auratus* (goldfish)	Schultz [39], Yu et al. [42], Shimizu et al. [52]
*Schizothorax* spp. (snowtrouts)	Mazik et al. [50]
*Synocyclocheilus* spp. (barbels)	Yu et al. [42], Rishi and Shashikala Rishi [43]
*Tor* spp. (mahseer)	J. Gui et al. [53]
*Zacco platypus* (freshwater minnow)	Yu et al. [42], Mazik et al. [50]
*Poecilia* spp. (guppy)	Schultz [39], Vriejenhoek et al. [44]
*Poeciliopsis* spp. (desert minnows)	Schultz [39]
*Protopterus dolloi* (slender lungfish)	Vervoort [54]
*Lepisosteus oculatus* (spotted gar)	Schultz [39]
*Stizostedion vitreum* (walleye)	Ewing et al. [55]
*Salmonidae* (salmons)	Allendorf and Thorgaard [56]
*Clarias batrachus* (walking catfish)	Pandey and Lakra [57]
*Heteropneustes fossilis* (indian catfish)	Pandian and Koteeswaran [58]
*Hyla versicolor* (grey treefrog)	Ptacek et al. [59], Mable and Bogart [60]
*Neobatrachus* spp. (burrowing frogs)	Mable and Roberts [61]
